# Traditional Games and Sports of the Women in the Kabylie

**DOI:** 10.3389/fpsyg.2020.614746

**Published:** 2021-01-28

**Authors:** Iman Nefil, Soraya Laaouad-dodoo, Pascal Bordes, Ahmed Torki

**Affiliations:** ^1^Laboratory I3SP (URP 3625), Institute of Sport-Health Sciences – University of Paris UFR/STAPS, Paris, France; ^2^Laboratory of Social Sciences, Department of Sociology, Salisbury University, Salisbury, MD, United States; ^3^Laboratory APSSES, Institute of Physical and Sports Education, Hassiba Benbouali University of Chlef, Chlef, Algeria

**Keywords:** Traditional games, Algerian society, women, culture, motor praxeology, socialization, Kabylie

## Abstract

With regard to the issue of the differences of the sexes, our study focuses on traditional games in Kabylie, taken and defined as part of the field of motor praxiology. A discipline inspired by the work of Pierre Parlebas. We will attempt through a field survey, to identify and interpret the meaning and distribution of the traditional games played by the women of Kabylie. From this perspective, the analysis of the internal logics of the practices are related to the system of interactions between players and their environment. This reveals valuable information on the structures underlying traditional games, and their ethno-motor characteristics as well as the world of ethnology of this vast mountainous region of Kabylie. By analyzing 92 traditional games played in the region of Kabylie allowed us to highlight on the one hand the cultural specificity, and on the other the richness of the socio-cultural aspects of this region. By examining how a game constitutes a cultural identity element of a geographical space, we explain the meaning of sport culture and identity of the Kabylie, by focusing particularly on the place of women and the indicators that reveal the relationships between men and women and distinguish the distribution of female and male roles. This also allows an understanding of the nature of traditional sports and their differentiation in terms of socialization between boys and girls.

## Introduction

Traditional games are an important part of the world’s recreational cultural heritage. Often confused with sports, the polysemous use of the two terms requires us to clarify the meaning attributed to each of the two terms. For Parlebas, a sports game is a: “Codified motor situation of confrontation, called” game “or” sport “by social authorities. A sports game is defined by its system of rules which determines its internal logic” (Parlebas, 1999). According to the author, the criterion of the institution invites us to distinguish two broad socially marked ludic categories: the group of institutional sports games consecrated by society “… and the class of traditional sports games ^∗^ neglected by the institution…” (Parlebas, 1999). While sport makes it possible to identify at the global level a vast set of cultural practices marked by a “standardization of practices” and by a “process of cultural homogenization,” traditional games reveal, at the local level “original cultural crucibles,” and “an exuberant ludo-diversity” (Parlebas, 2016).

For this purpose games is an activity that deserves attention. Far from being unproductive, sterile, it is in fact an element of great importance in the sense that it “deeply engages the player, the team and the company in a motor creation carrying a meaning” (Parlebas, 1990, p.70). Games carry a cultural identity, which leads us to say that sports games appear as the mirror of society. Based on the thesis according to which games bear witness to the ethnomotricity of a people (Parlebas, 1999, p. 145), we will attempt to understand traditional games in their sociological meaning and to define them in the context of Kabylie society.

## Traditional Games in Algeria: State of Place

Studies of traditional games in Algeria, although few, all reveal that traditional games is not separable from culture if we want to understand the institution of Algerian society. In this regard, (Kharchi, 1978) has identified and described some traditional physical games in the Setif region, but the author has only quoted the games without studying them as a separate field. In her doctoral work (Laaouad-Dodoo, 2007) conducted a study on 92 traditional sports games in the Kabylie region. Just as (Mellal-Bounaas, 2007), has listed 93 games of Tuareg that have been practiced in the city of Tamanrasset. In the extreme south of Algeria, and another more recent dedicated for the Mozabites games made by Bekai (2017), confirm a close link between the structures of the games and the social structures of the different groups studied.

In the Kabylie region, we are in the same situation as that transcribed by Boulifa (2003), which offers a description of more than twenty games played by the children of Grande Kabylie at the beginning of the 20th century. The interest of these games lies both in their diversity, in the attention given to them by early childhood, in the precision of their description and in the link, that Boulifa often makes with the socio-cultural context. The main limitation is that it is almost exclusively boys’ games, that is, games played only by boys.

In the absence of bibliographical elements on our problem, we can rely on more general sociological data. Thus, according to Pierre Bourdieu, the social structure of Kabylie and differentiated socialization are based on bipartisanization (Bourdieu). A division of roles and spaces into a private world, represented by the sacred, which refers to the female and an open public world outside, belonging to the male space. In this context, what could a praxiological analysis of traditional games produce as knowledge about the process of sexual socialization in Kabylie society?

To answer our question, we put forward the following hypotheses:

- Traditional women’s games are closely related to the socialization of Kabylie women by the fact that girls play alone and do not mingle with boys.
- Traditional games such as a system of interaction and communication with others, space, time, and objects, are in collusion with the model of social organization and representation of Kabylie culture.

## Materials and Methods

In a first phase, we carried out a bibliographical research showing the traditional games of Kabylie. At the same time, we undertook a field survey, which consists in collecting the games using classic sociological tools such as: participant observation, field notes, interview semi-directive where individual had the freedom to describe the game and their function.

The field investigation consisted of conducting interviews based on a game observation sheet developed by Pierre Parlebas. It is a descriptive grid whose variables relate to data (spatial, material used, game flow, game structure, and player behavior). (Parlebas, 1990, p.192–p.212). These interviews are semi-direct, and the questions are extracted from the observation sheet prepared by Parlebas (1990). They are conducted with people from the observed village, and supplemented with observation of their daily lifestyle. The collection of traditional games, which consists of exhuming the ancient games of memories, sometimes exposes to mockery and smiles. Such a lack of knowledge and the discredit of the game have only made us more tenacious and determined to continue our search, to reveal its richness.

From the materials thus collected, we opted for an analysis centered on Parlebas’ sports game theory. This refers to the study of the internal logic of observed practices, defined by Parlebas as a system of relevant features of a motor situation and which manifests itself in all forms of relationships with others, with space, object, and time (Parlebas, 1981).

The standard analytical sheet played a primordial role in the methodological process of data analysis, on the one hand because it collects the identifying features of the internal logic of each play situation, on the other hand because it simplifies the deciphering and interpretation of the results. We thus have ninety-two cards that are like identity cards for the games studied.

In all, our analysis covered a corpus of 92 games that were submitted for analysis, which allowed us to classify the play activities and distribute them over the eight categories of the classification of motor situations used in praxiology.

Finally, and to highlight the socio-cultural aspects of the games, we used the crossover of data from the internal logic of these games and those of the “external logic” that attributes new or unusual symbolic meanings to it (Parlebas, 1981). This external logic is none other than the set of socio-cultural relationships that reveal the social representations and symbols experienced in the games.

## Results

**FIGURE 1:**
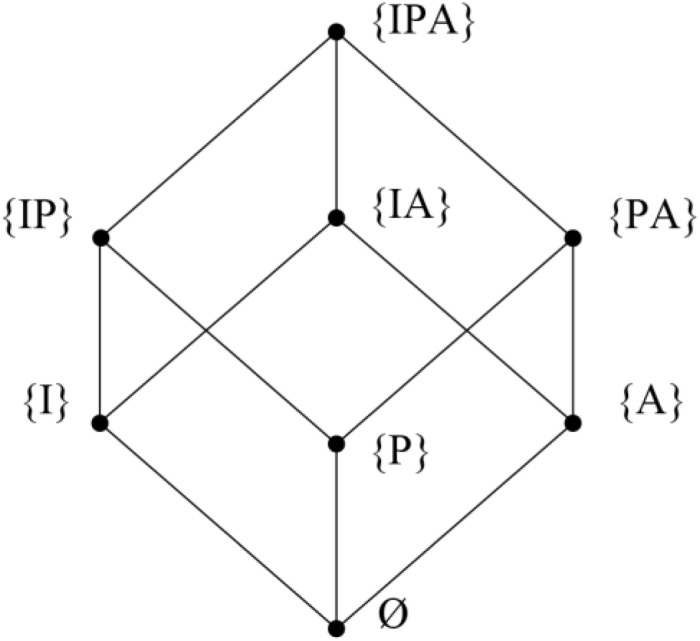
Classification of traditional sportive games according to the S3 simplex. These are the set of eight parts of the (IPA) set organized by the inclusion relationship. This simplex was chosen because it allows a better visualization of the resources of this classification. (I): The intervention of an uncertainty due to the interaction with the material environment, (P): the intervention of an uncertainty born from the interaction with partner (s) and (A): the intervention of an uncertainty created by the interaction with opponent (s).

The distribution of ninety-two sportive games from the Kabylie region according to the classification named “S3 simplex” makes it possible to consider the results of the Figure 1.

### Some Significant Results of Traditional Sports and Games in Kabylie

**FIGURE 2:**
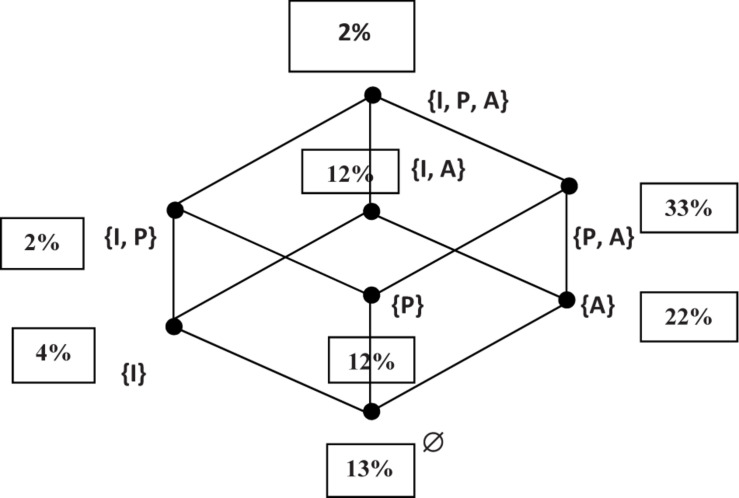
Distribution of ninety-two games in the Kabylie region according to the S3 simplex.

According to the Figure 2 we note that among the eight categories of the classification, (33%) of the games played belong to the class (P, A), games in the presence of partners and opponents in a stable environment. (13%) are solo games and without uncertainty of the environment (category “Ø”). Among these games, we can cite as an example the game Redjma (Target shooting) which is played during major events (parties, weddings, circumcision, ceremonies, etc.). Group games are more numerous (35%) than individual games (17%), games of strict opposition are more numerous (34%) than games of strict cooperation (14%). On the other hand, the games in environments with uncertainty are much reduced; 20% compared to 80% games in semi domestic space.

### Distribution of Games According to the “Sex” Criterion

**FIGURE 3:**
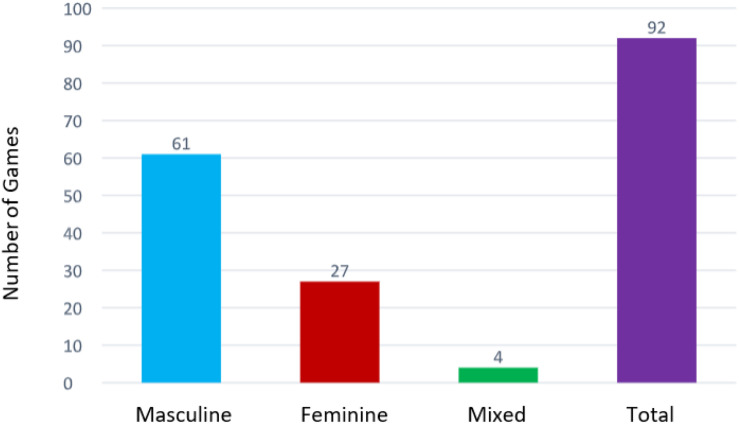
Distribution of games by sex.

We note in Figure 3, that more than two thirds of sports games are male games, 66% as opposed to 29% of female games. Mixed games hold the minimum portion of 4%. This clear superiority of male practices over mixed practices confirms the separation of the sexes in Kabylie society. Man and woman belong to two distinct worlds. We could also explain this observation by the age factor. Girls stop playing at the age of 12. Beyond this age, they prepare for the life of a woman. While the boys continue their playing activity beyond the age of 16.

The separation of the games is due to the sexual division. The division of space and the division of labor, even if it is not rigid, are opposed to mixed sex as Camille Lacoste-Dujardin states in the following words:. “They don’t have the same habit, the same custom, and do not have the same thinking. They don’t even speak the same language anymore.” (Lacoste-Dujardin, 1970, p.65). To the question: “Why don’t you play with the boys?” One girl explains: “We only play with girls because it is shameful to play with boys.” The term “shameful” indicates the notion of honor (Hurma), which according to Bourdieu means the whole of what “haram” is to say prohibited, in short the sacred, to which every person must be attached by- above all, as a precious and cherished value (El azz). To play with the other sex is to break the Hurma, which is a fundamental moral of Kabylie life. In terms of values, men are required to maintain the Hurma’s strength and safeguard their family and land, and women are to protect the honor of the group.

Pierre Bourdieu points out that this sexual division is a territorial division: “Clear distinction between the female space, the house and its garden, place of excellence of the Haram, closed space, secret, protected from intrusions, and looks. The male space, the Thajma’th, place of assembly, the mosque, the café, the fields, or the market. On the one hand, the secret of intimacy all veiled in modesty. On the other, the open space of social relations, political, and religious life” (Bourdieu, 1972, p.9).

#### The Space of the Game: Between the Female Domestic Space (the house) and the Male Public Space “Tadjmaat” (External Environment)

With regard to space, the games of the children of Kabylie take place in semi-domestic places (75%). This is neither a very specific nor a totally wild environment. It is an unmarked, improvised space, often flat and regular. It is only during early childhood that boys and girls engage in joint games. This often consists of ludo-motor games without rules such as sliding games, or olive picking, or the ritual of the rain “game called: Boughandja” where the children are escorted by an old lady and go around town. After this early childhood period, as Camille Lacoste-Dujardin points out, “… boys and girls stop seeing each other. Their destinies, differentiated from their birth but mingled in the same maternal intimacy and common games, can no longer at this age merge and separate radically” (Lacoste-Dujardin, 1970, p.65).

Girls play near the houses, in the large courtyard (El lhara) which brings together the houses of a group of families, at the fountain, or in the fields under the supervision of the mothers. On the other hand, we see that the boys play at the exit of the village, at the oued (the river) or at the meadow. These places allow better hiding and better movement, especially in the case of kora (ball) or chase games. Especially since we know that the alleys in Kabylie are very narrow given their mountain topology. A young boy Kabyle explains: “We avoid playing near houses so as not to disturb the old people.” To disturb the old people is to disturb the house, which is a sacred place, and to violate this Horma space. In addition, the statute of the old men, the *imraren*, is much respected in Kabylie. They are the guardians of the *nif*, of the honor of the village. Idle, sitting in the public square, and they watch the comings and goings of passers-by. The street is not the territory to play, it is not anonymous, it is the place where you check passers-by, and find out if there is a stranger by any chance. Just as the space of the house is sacred and subservient to an invisible world that must be respected, this choice of places is subject to cultural reasons. This separation of spaces must be understood in the sexual division. For girls, the space of the game is that of places reserved for women (the courtyard and the fountain). On the other hand, boys, considered as males, must join the male clan. They are forbidden to play near the houses or else they will break *Horma*, the honor of the family. In addition, distant games in nature are very rare in Kabylie. The mountain constitutes the Kabyle landscape overwhelmingly; however, games are rare in these wild places because the Kabyles believe that these places are inhabited by a harmful invisible world. As Lacoste-Dujardin notes: “A mountain of stone riddled with cracks and caves that are difficult to access, it is of course the natural home of geniuses of all kinds and of snakes, also called *Teryel*” (Lacoste-Dujardin, 1970, p.136).

Our results show that games in flat spaces represent 75% of all games, compared to 25% in rough locations. This clearly means that children prefer to play in flat and large places, where they can hide and move around easily. It should also be noted that playing in a neutral and sacred space between two villages, often near a sanctuary, promotes the competitive spirit of the players.

### The Relationship to Others

It turns out that the traditional sportive games universe of Kabylie children has a tendency for group games, common games, where the player is in constant contact with other situations of “sociomotricite” This then gives rise to a meeting, to “exchanges” (passes, interception, strikes, tackles, calls, and supports). These games, in which others bring uncertainty and conditions the action of the game, correspond to 77% of the corpus of our traditional games and are therefore strongly representative. Sociomotor games clearly favored (83%) compared to psychomotor games on solo (17.38%). Opposition games where adversity flourishes and oppositions group together 70% against 51% of solidarity activities compared to duels. We can identify two main classes of duels: duels, which exclusively pitch two players (*Tikare*) which resemble the sport of karate, and team duels *(kora*) el *khateime* (the ring).

If socio-motor games are predominant, it is because in Kabylie, the group has a major weight and the work in the fields for men and women is carried out in groups. We see this community in the case of women who go to the fountain in groups of relatives and neighbors to carry water, and who also help each other to lift and put on their heads the huge sheaves from the harvest and also for collecting and also for collecting olives in autumn.

### The Function of the Relation to Material

In most cases, games require the presence of material. It can be something to throw, grab, or any object that can be crafted. Among all the traditional games, we rely on the entire Kabylie corpus collected, 33% of games are played without material, 31% use objects borrowed from the domestic environment and only 14% use material from the natural environment. Generally, the play objects from the domestic environment are the “jug, the fez, the scarf, burnous, the ball, the gun, the reel, the spinning top, and the eggs. Some of these objects are made by the players like the rifle, the reel and the ball.” In addition, olive and fig trees play a preponderant role as a natural environment from which the Kabyles draw to make their play objects: olives, branches, sticks, eucalyptus leaves, prickly pear leaves, pebbles… These natural objects are treasures for children who use them to make balls (Koura), sticks (Matreg), guns, knucklebones or stones. In his book “Kabylie Côté Femmes” (Laoust-Chantréaux, 1990, p.67), indicates that the objects, which correspond to domestic life or agricultural work, are of family and regional manufacture and divided by the sexes. Pottery is still made by women. The relationship to the object in the kabylie ludo- culture is a cultural relationship. Girls often use the scarf to blindfold the game of hide and seek. In the rainy season, when the earth is malleable, they play in the mud, dig their legs and play around. The jug is also used in girls’ games.

### The Function of the Relation to Time

What does the inclusion of the “time” variable reveal? Two-thirds of the 69% games do not have a point-based system to determine the winner of the game. Games are not sanctioned by a result. We see that 26% of them take place with a limited score. These score-limit games are not timed out. These are indeterminate games that end with players’ fatigue or when they decide to stop playing. Many traditional games are played around the agricultural seasons. This observation is important to take into account from the point of view of the games. The life of the Kabylie peasants is dominated by agricultural work in the fields and the cultivation of gardens. These seasons as determining symbols of time are inserted in the agrarian calendar. In summer, there are fighting games, agonistic games between two villages. At the start of the fall, we play more of the individual game, (spinning top, pushing a cork disk). In spring, the favorite game is the game “*chemcham* leapfrog” whose symbol corresponds to the rites protecting the fertility of the flock. In Great Kabylie, the same season the shepherds practice the game *(tahjart*) or “*nnif*” which consists in knocking down a tile placed in balance with stones. This ritual game is also practiced on weddings. As Danièle Jemma-Gouzon writes: “In these ancient peasant societies which have over time a conception of circular and global advantage rather than linear and unfolded rather internalized than externalized, it is the ancestors who define the past, but also make the present and secure the future” (Jemma-Gouzon, 1989, p.77). The Kabyle child grows up to learn to interpret time signals, based on practical games according to the seasons.

## Discussion

### The Cultural Identity of the Kabylie Games

After all these data, we understand that games are carriers of Kabylie culture and socialization in addition to sexual division. From our corpus of games, it turns out that girls’ games tend to be circle games, approach games (hide and seek), or “carry” games (carrying on the back, on the shoulders, carrying one of the players from one place to another). If the games of “carrying” are practiced a lot by girls, it is because the Kabylie woman from an early age learns to carry a jug of water on her head. This makes it possible to develop her back muscles, which allows her to stand up straight. Through these early games, the Kabylie girl incorporates these values into her body through play actions.

Boys’ games, on the other hand, are mostly ball games, fighting games, or physical skill like shooting at the target. There is a clear superiority of physical games where motor skills are in full swing. Physical energy is everywhere. In men’s games, the player’s body is the target of the game 35 vs. 31%, whose target is material. These are games where the objective is to reach a goal such as Kora games, football or archery *(Radjama*). When the body is the target, we often see violent games, which often expose the players to beatings and injuries such as *(tikare* game), where the players kick each other, or the “sheep game.” “Blind” in which the player, blindfolded, receives blows from the opponents with the (glouza) the beret of the burnouses. It would seem that the ludic relationship within traditional games refers to the cultural values favored by the Kabylie people, and in particular to the strength to defend their group, their land and their village. The social function of traditional games is fundamental to the socialization of the child, even to make it conform to standards and models of attitudes. One way to internalize the community’s secret standards systems.

## Conclusion

Traditional female games are closely linked to the socialization of the Kabylie woman. System of interaction and communication, they are in collusion with the model of social organization and representation of Kabylie culture and participate in the socialization of the younger generations. From an early age, the girl was removed from male influence. “We weave between her and men a veil of shame, which will not be torn until marriage” (Zerdoumi, 1982). From the age of six, Nefissa Zedoumi tells us, that from 6 years old, the little girl mixes less and less with boys of her age. Girls start to feel confused around their older brothers and respect the prohibition on playing with boys.

In traditional rural areas, the majority of Algerian families, especially mothers, do not educate boys and girls in the same way; there is a favoritism toward the male sex, which creates a complex in the girl.

We find the same similarity in the analysis of sport by Hargreaves (1994), p.6, 7, which states that this is an inherently controversial matter, and sociology incorporates different and contradictory theories of society. Those, which in general support conventional ideas about sport, about the nature of society, and on male and female identities; and those who question them. As a result, we can see that the history of the sociology of sport reflects the long history of male dominance in modern sports and dominant ideas about sexual difference. The history and sociology of sport reflect the male dominance of academic discourse (Hargreaves, 1994, p. 7).

In traditional society, women are educated for marriage. For her, it is a matter of giving birth to male children preferably, of serving her family, then her husband and her in-laws. It is only once she is a stepmother that she will have the right to be respected.

Ludic practices is far from pure frivolity “participates in the cultural identity of each community, which thus stages original ludic- scenarios intimately linked to its own lifestyles, beliefs and passions” (Parlebas, 2005, p.6). In this sense, traditional motor games are part of this socialization, which tends to prepare Kabylie girls for their future role as women as their society conceives it.

## Data Availability Statement

The raw data supporting the conclusions of this article will be made available by the authors, without undue reservation.

## Ethics Statement

Ethical review and approval was not required for the study on human participants in accordance with the local legislation and institutional requirements. Written informed consent from the participants was not required to participate in this study in accordance with the national legislation and the institutional requirements.

## Author Contributions

IN and SL marked the design of the article. SL carried out the field survey and analyzed the data. IN wrote the first draft of the manuscript. IN, SL, PB, and AT organized the database, interpreted the results, and wrote the sections. All cited authors have made substantial, direct, and intellectual contributions to the work and approved for publication.

## Conflict of Interest

The authors declare that the research was conducted in the absence of any commercial or financial relationships that could be construed as a potential conflict of interest.
